# Effectiveness of Cyclic Voltammetry in Evaluation of the Synergistic Effect of Phenolic and Amino Acids Compounds on Antioxidant Activity: Optimization of Electrochemical Parameters

**DOI:** 10.3390/foods13060906

**Published:** 2024-03-16

**Authors:** María José Jara-Palacios, Emilio Begines, Francisco J. Heredia, María Luisa Escudero-Gilete, Dolores Hernanz

**Affiliations:** 1 Department of Analytical Chemistry, Facultad de Farmacia, Universidad de Sevilla, 41012 Sevilla, Spain; mjara@us.es (M.J.J.-P.); emibegher@gmail.com (E.B.); 2 Food Colour and Quality Laboratory, Facultad de Farmacia, Universidad de Sevilla, 41012 Sevilla, Spain; heredia@us.es (F.J.H.); gilete@us.es (M.L.E.-G.)

**Keywords:** cyclic voltammetry, phenolic compounds, amino acids, electrochemistry, antioxidant activity

## Abstract

Antioxidant activity can be evaluated using cyclic voltammetry (CV). The aim of this work is to verify the efficacy of CV in evaluating the synergistic effect of bioactive compounds, such as phenolic and amino acid compounds, on antioxidant activity. Therefore, three types of model solutions were prepared: individual model solution (phenol and amino acid), (b) binary model solutions (phenol-phenol and amino acid-amino acid) and (c) mixed phenol–amino acid solutions. Electrochemical measurement conditions were optimized for phenolic compounds (pH 3.0, 1.0 g/L and 100 mV/s) and for amino acids (pH 7.0, 2.0 g/L for amino acids and 100 mV/s), and, for each solution, the functional groups responsible of the anodic and cathodic peaks were established. The peak anodic potential (E_pa_) and the onset potential (E_on_) were two parameters of great importance. The first one was used to classify the solutions according to their antioxidant potential. In general, all the binary and mixed solutions had lower values of E_pa_ than the corresponding individual model solution, which indicates an improvement in the antioxidant potential. The second one was used to evaluate the synergistic effects of phenolic compounds and amino acids.

## 1. Introduction

Electrochemical techniques are used to study chemical reactions that involve electron transfers and that entail the oxidation or reduction of a compound. These techniques allow for simple and fast measurements and are relatively inexpensive. Electrochemical methods can allow for the selective detection of chemical compounds with good sensitivity and responses, although outputs are sometimes difficult [1]. Cyclic voltammetry (CV) is an electrochemical technique that is based on the current-potential response of a polarizable electrode in the analyzed solution [2]. Cyclic voltammetry provides a measure of redox potential, which is used to describe the overall reduction or oxidation capacity of a compound and has been used to study the electrochemical behavior of different antioxidant compounds [3].

Antioxidants reduce oxidative stress because they delay or avoid the oxidation of macromolecules in living organisms due to their antiradical properties. In addition, antioxidants also prevent oxidative processes in foods. In recent years, consumers have become more aware of the importance of taking foods containing antioxidants that influence health. In this sense, the food industry is exhibiting much interest in using antioxidants in food processing to improve the bioactivity of foods.

The antioxidant activity of foods rich in antioxidant compounds, such as wine, milk, honey, grape, kiwi, tea, and coffee, has been evaluated using cyclic voltammetry [4,5,6,7,8,9,10]. Among the antioxidants present in foods are phenolic compounds and amino acids, whose redox potential can be evaluated by cyclic voltammetry [1,11,12,13]. Although the antioxidant potential of some phenolic compounds and amino acids has already been evaluated, no joint evaluation of both compounds by voltammetry has been performed.

Considering the importance of these compounds (phenolic compounds and amino acids) in foods, synergistic antioxidant activity could be interesting for the food industry. Concretely, these compounds and their synergistic effect on antioxidant activity are of great importance in the wine industry. On the one hand, wine has beneficial effects on health related to phenolic compounds. Furthermore, the constituent amino acids of antioxidant peptides, which could exert positive effects on the redox status of the wine matrix, could potentially act as protective agents against oxidation. The results of one of our research projects indicated that proteins could increase the antioxidant capacity of wine and, thus, the stability of phenolic compounds, such as anthocyanins, preventing their degradation. In addition, results indicated that, although in moderate proportions, grape seed proteins also contain aromatic amino acids and proline, which could play a relevant role in copigmentation reactions in which phenolic compounds such as flavonols and anthocyanins are involved. These bioactive compounds can be naturally present in the wine or can be added externally during the winemaking process, improving the quality of the wine.

Therefore, the purpose of this work is to verify the efficacy of cyclic voltammetry in evaluating the synergistic effect of bioactive compounds, such as phenolic compounds (hydroxycinnamic acids, benzoic acids, and flavonoids) and amino acids, on antioxidant activity. Both types of compounds are important in wine. For this purpose: (1) a voltammetry method has been developed by optimizing the experimental measurement conditions in model solutions: pH (3.0 and 7.0), concentration (0.5 and 1.0 g/L for phenolic compounds and 1.0 and 2.0 g/L for amino acids) and scanning rate (50 and 100 mV/s); (2) the electrochemical behavior has been evaluated with the optimized voltametric method in three types of model solutions: (a) individual model solution (phenol and amino acid solutions), (b) binary model solutions (phenol-phenol and amino acid-amino acid solutions) and (c) mixed phenol–amino acid solutions.

## 2. Materials and Methods

### 2.1. Standards and Reagents

Gallic acid, (+)-catechin, (−)-epicatechin, ferulic acid, caffeic acid, syringic acid, vanillic acid, quercetin, quercetin 3-*O*-rutinoside, cysteine, histidine, tyrosine, and tryptophan were purchased from Sigma-Aldrich (Madrid, Spain). Methanol, ethanol, hydrochloric acid, acetic acid, sodium acetate, sodium dihydrogen phosphate, and disodium hydrogen phosphate were of analytical grade (Panreac, Barcelona, Spain).

Stock solutions of the phenolic compounds and amino acids were prepared separately. The concentrations of the working standards were 0.5 and 1.0 g/L for phenolic compounds and 1.0 and 2.0 g/L for amino acids. For binary and mixed model solutions, the concentration was 1.0 g/L for the phenolic compounds and 2.0 g/L for the amino acids.

### 2.2. Cyclic Voltammetry Measurement

The experimental configuration for recording cyclic voltammograms consists of an electrochemical cell and three electrodes, all immersed in a liquid and connected to a potentiostat. The potentiostat AUTOLAB model PGSTAT 302 N (Metrohm-Eco Chemie, Utrech, The Netherlands) is controlled by General-Purpose Electrochemical System (GPES) Nova 1.11 software. The conventional three-electrode system consisted of a glassy carbon working electrode, a platinum auxiliary electrode, and an Ag/AgCl reference electrode (Metrohm-Eco Chemie, The Netherlands).

Before measurements, the working electrode was polished in an alumina/water suspension, rinsed with Milli-Q water, and sonicated for 2 min. The electrolyte solution was transferred to a glass water jacket electrochemical cell (EG&G, Princeton, NJ, USA) connected to a circulator that held the sample temperature at 25.0 ± 0.5 °C. The solution was deaerated with an inert gas (N_2_) for 10 min, and after one minute, a running scan was taken. The cyclic voltammogram scans were performed from 0.0 to 1.0 V for model solutions with phenolic compounds and from 0.0 to 2.0 V for model solutions with amino acids and mixed solutions at a scanning rate of 50 and 100 mV/s. The electrochemical parameters extracted from the cyclic voltammetry curve were the anodic peak current and potential (I_pa_ and E_pa_, respectively), the cathodic peak current and potential (I_pc_ and E_pc_, respectively), the potential mid-way between the anodic and cathodic peaks (E°) calculated from ½ (E_pc_ + E_pa_) and ΔE calculated from E_pa_ − E_pc_.

Two buffer solutions were tested for measurements. The first solution was phosphate buffer at pH 7.0 composed of 65% (*w*/*v*) 50 mmol/L disodium hydrogen phosphate and 35% (*w*/*v*) 50 mmol/L sodium dihydrogen phosphate [2], and the second solution was acetate buffer at pH 3.0 composed of sodium acetate 0.1 mol/L with added acetic acid.

For electrochemical measurements, 1 mL of the model solution (individual, binary, or mixed) was diluted with 25 mL of buffer solution. All solutions were prepared in triplicate, and each solution was recorded in triplicate.

### 2.3. Statistical Analysis

One-way analysis of variance (ANOVA) was applied to determine whether significant differences exist among the electrochemical behavior of the individual model solution, binary model solutions, and mixed phenol–amino acid solutions. A statistically significant level was considered at *p* < 0.05. These statistical analyses were performed using StatSoft Statistica^®^ V 8.0 software.

## 3. Results and Discussion

### 3.1. Electrochemistry of Phenolic Compounds

CV was used to monitor the electrochemical properties of the three groups of phenolic compounds: hydroxycinnamic acids, benzoic acids, and flavonoids. Specifically, caffeic and ferulic acids (hydroxycinnamic acids), gallic, syringic and vanillic acids (benzoic acids) and catechin, epicatechin, quercetin 3-*O*-rutinoside and quercetin (flavonoids), were monitored.

First, the electrochemical conditions have been optimized in gallic and caffeic acids, catechin and quercetin 3-*O*-rutinoside. Subsequently, with the optimized conditions, the electrochemical behavior of nine individual model solutions and the electrochemical behavior of 36 binary model solutions (with two phenolic compounds) were measured.

#### 3.1.1. Optimization of Electrochemical Conditions

First, conditions such as pH, concentration and scanning rate for cyclic voltammetry measurement were studied in four phenolic compounds (gallic and caffeic acids, catechin, and quercetin 3-*O*-rutinoside) for optimization.

Electrochemical measurements were taken at different pH (3.0 and 7.0), phenolic concentrations (1.0 and 0.5 g/L) and scanning rates (100 and 50 mV/s). Table 1 shows the electrochemical parameters (E_pa_, E_pc_, I_pa_, I_pc_, E°, and ΔE) extracted from the cyclic voltammetry curves of the phenolic compounds. Different conditions show significant effects (*p* < 0.05) among electrochemical behaviors. Specifically, pH differences in the solutions showed a significant effect on I_pa_, E_pa_, and E°, as well as differences in the scanning rate on I_pa_ and I_pc_.

**Figure 1:**
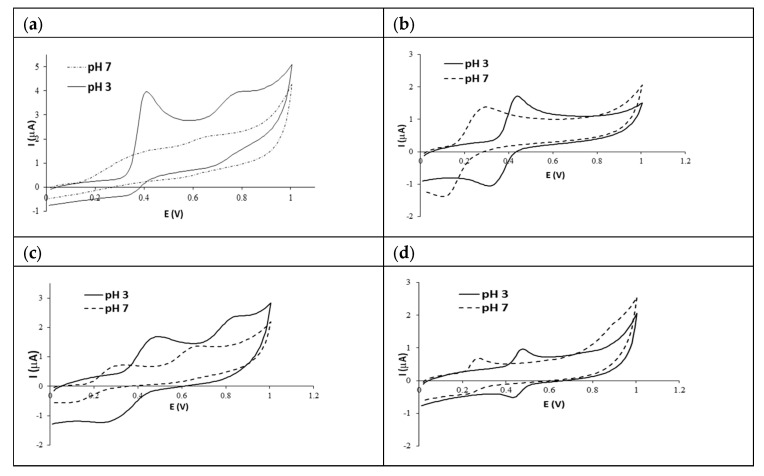
Voltammograms of gallic acid (**a**), caffeic acid (**b**), catechin (**c**), and quercetin 3-*O*-rutinoside (**d**) (1.0 g/L and 100 mV/s): pH 3.0 (acetate buffer of sodium acetate 0.1 M with added acetic acid to give a pH) and pH 7.0 (phosphate buffer of 65% (*w*/*v*) 50 mM disodium hydrogen phosphate and 35% (*w*/*v*) 50 mM sodium dihydrogen phosphate).

Gallic acid, adjusted to pH 3.0, showed two well-defined anodic peaks (E_pa1_ = 0.414 V and E_pa2_ = 0.786 V) and one cathodic peak (E_pc1_ = 0.350 V). The last cathodic peak does not appear at pH 7.0 (Figure 1a), indicating that at this pH, the oxidation product is not reduced on the glassy carbon electrode [14].

Furthermore, the E_pa_ values at pH 7.0 are displaced with respect to pH 3.0. Yakovleva et al. (2007) [15] observed that an increase in the pH of the electrolytic solution leads to a decrease in the E_pa_ value, caused by a decrease in the degree of antioxidant protonation and a change in the charge of the molecule to negative values. These pH differences in the electrolyte solutions also showed significant differences (*p* < 0.05) on the other electrolyte parameters: E_pc_, E°, and ΔE values of the first peak and E_pa_ values of the second peak.

Additionally, considering the gallic acid concentration (1.0 and 0.5 g/L) and the scanning rate (50 and 100 mV/s) for the measurement of cyclic voltammetry, no significant changes in E_pa_ and E_pc_ values were observed. The I_pa_ value increases with increasing gallic acid concentration and scanning rate, but this relationship is not linear when higher concentrations of phenolic are used [1,16].

The electrochemical behavior of caffeic acid at different pH, concentrations, and scanning rates is shown in Table 1. As can be seen in Figure 1b, the voltammogram corresponding to caffeic acid at different pH levels shows a single anodic peak with a corresponding cathodic peak, which is related to the mechanism of oxidation of catechol groups. Significant differences (*p* < 0.05) were found in the potential values of the anodic and cathodic peaks (E_pa_ and E_pc_) and the potential midway between the half peak potential (E°) according to the pH solution. The E_pa_ values depend on the pH of the electrolyte solution, which changes from 0.439 to 0.301 V at pH 3.0 and pH 7.0, respectively. These potential values are consistent with previous studies, considering the expected 59 mV per pH unit shift in the potential [17].

The concentration of caffeic acid (1.0 and 0.5 g/L) did not show significant differences in the values of the electrochemical parameters studied. Regarding the scanning rate for the measurement of cyclic voltammetry (100 and 50 mV/s), significant differences (*p* < 0.05) are observed in I_pc_, being higher at 100 mV/s. 

The same results were obtained for catechin; the pH of the electrolyte solution changed significantly (*p* < 0.05) in terms of electrochemical behavior, mainly concerning the values of the first anodic and cathodic peaks potentials (E_pa_, and E_pc_) and the potential midway between the half peak potential (E°) (Table 1). The catechin voltammogram shows two main anodic peaks at 0.495 and 0.835 V and one cathodic peak at 0.261 V at pH 3.0, while at pH 7, these potentials are displaced at lower values (E_pa1_ = 0.334 V, E_pa2_ = 0.681 V, and E_pc1_ = 0.107 V) (Figure 1c). Regarding catechin concentration (1.0 and 0.5 g/L), only significant differences (*p* < 0.05) were found in the ∆E values, providing information on the number of electrons transferred in a reversible redox reaction. The scanning rate (100 and 50 mV/s) for the measurement of caffeic acid did not show significant differences in the values of the electrochemical parameters. 

The electrochemical behavior of quercetin 3-*O*-rutinoside at different pH, concentrations, and scanning rates is shown in Table 1. As can be seen in Figure 1d, the voltammogram corresponding to quercetin 3-*O*-rutinoside at different pH levels shows two anodic peaks and one cathodic peak. Significant differences (*p* < 0.05) were found in the potential values of the first anodic and cathodic peaks (E_pa_ and E_pc_) and the potential midway between the potential of the half peak (E°) according to the pH solution. In acid solutions, the potential value of the anodic and cathodic peak increases; therefore, at pH 3, the E_pa1_ and E_pc1_ values were 0.479 V and 0.431 V, while at pH 7, they were 0.277 and 0.212 V, respectively. The concentration of quercetin 3-*O*-rutinoside and the measurement scan rate did not show significant differences in terms of the values of the electrochemical parameters studied. 

From the results obtained, it is concluded that the E_pa_ and E_pc_ values are mainly modified by pH, whereas the concentration of the electrolyte solution and the scanning rate affect the I_pa_ and I_pc_ values. In this regard, the values of E_pa_ and E_pc_ depend on the nature of the electrochemical compounds (qualitative analysis), while those of I_pa_ and I_pc_ are measures of the concentration (quantitative analysis). Based on the results obtained, the measurement conditions selected were as follows: pH 3.0, 100 mV/s and 1.0 g/L. 

#### 3.1.2. Electrochemistry of Phenolic Compounds: Individual Model Solutions

The electrochemical behavior of nine phenolic compounds belonging to different phenolic groups has been evaluated under previously established conditions. Table 2 shows the electrochemical parameters extracted from the voltammograms of the individual model solutions.

Gallic, syringic, and vanillic acids were selected as hydroxybenzoic acids. The voltammograms obtained for the three compounds present two anodic peaks and one cathodic peak. However, despite the structural similarity of these three phenolic compounds, the presence of methoxyl groups in the aromatic rings of syringic and vanillic acids makes their oxidation mechanism differ from that of gallic acid. For gallic acid, the first anodic peak and its corresponding cathodic peak are related to a characteristic reversible reaction of the –OH groups at positions 3 and 4 of the aromatic ring [2]. For the other two acids, the first anodic peak and the cathodic peak are associated with the transfer of an electron, giving rise to the formation of the corresponding phenoxy radical. The second anodic peak is associated with a second transfer of an electron, giving rise to a carbocation that simultaneously generates the corresponding dioxobenzoic acid and a molecule of methanol via hydrolysis [18,19].

With respect to hydroxycinnamic acids, caffeic and ferulic acids were evaluated. The caffeic acid shows an anodic peak and a cathodic peak, which are related to the oxidation of the catechol group. However, the ferulic acid shows two anodic peaks and one cathodic peak. The first anodic peak obtained for ferulic acid is associated with the transfer of an electron to give rise to the corresponding phenoxy radical [20]. The second anodic peak is associated with a second transfer of an electron that would give rise to a carbocation that, via hydrolysis, generates 3,4-dioxocinnamic acid and a molecule of methanol [20]. 

**Figure 2:**
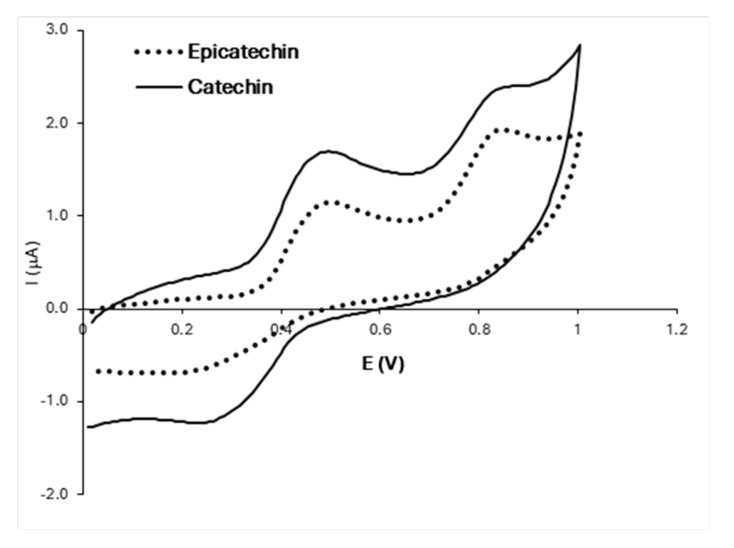
Voltammograms of catechin and epicatechin under optimized conditions (pH 3.0; 1.0 g/L and 100 mV/s).

Catechin and epicatechin show great similarity in electrochemical behavior due to their nearly identical chemical structures and the fact that they undergo the same oxidation mechanism (Figure 2). The first oxidation peak appears at 0.495 V in both voltammograms and corresponds to the oxidation of the catechol group. The cathodic peak, corresponding to the reduction of the quinone group, appears at 0.261 V for catechin and 0.148 V for epicatechin. The second anodic peak appears at 0.835 V for catechin and 0.851 V for epicatechin and represents oxidation of the resorcinol group [21]. However, some authors indicate that this anodic peak may correspond to the irreversible oxidation of the –OH group at position 3 of the non-aromatic ring [14,22].

The voltammograms of quercetin and quercetin 3-*O*-rutinoside show two anodic peaks and one cathodic peak. Both compounds undergo the same oxidation mechanism: The first anodic peak and its corresponding cathodic peak correspond to the reversible oxidation of the catechol group. The second anodic peak represents the oxidation of the resorcinol group. 

E_pa_ values of the first peak in the anodic scan were used to classify the compounds according to their antioxidant potential. A compound with a lower E_pa_ value is a better antioxidant because it has a greater ability to donate electrons [23]. Therefore, the E_pa1_ order is as follows: gallic acid (0.414 V), caffeic acid (0.439 V), quercetin (0.439 V), syringic acid (0.447 V), ferulic acid (0.471 V), quercetin 3-*O*-rutinoside (0.479 V), vanillic acid (0.487), catechin, and epicatechin (0.495 V). Taking this order into account, gallic acid is the most potent antioxidant. With respect to phenolic acids, gallic acid is a better antioxidant than syringic acid and vanillic acid. Among flavonols, quercetin shows a higher ability to donate electrons than quercetin 3-*O*-rutinoside. Catechin and epicatechin present the same ability. Taking into account the second oxidation peak, the E_pa2_ order is as follows: syringic acid (0.673 V), ferulic acid (0.754 V), and quercetin 3-*O*-rutinoside (0.754), gallic acid (0.786 V), vanillic acid (0.835 V) and catechin (0.835 V), epicatechin (0.851 V) and quercetin (0.851 V). In this case, syringic acid undergoes the easiest oxidation [23].

#### 3.1.3. Electrochemistry of Phenolic Compounds: Binary Model Solutions

A total of 36 binary model solutions, with two phenolic compounds, were prepared to study the possible interaction between the oxidation processes of different compounds. Table 2 shows the different combinations with their corresponding electrochemical parameters. As can be observed, all of the mixtures retained their two anodic peaks, but in general, the E_p_ values were different for a compound alone or mixed.

Taking into account the E_pa1_ values, gallic acid was a better antioxidant alone than in combination; however, the other eight compounds are easier to oxidize when mixed with any other compound than alone (Table 2). 

Syringic, vanillic, caffeic, and ferulic acids, catechin, epicatechin, and quercetin 3-*O*-rutinoside mixed with quercetin have an E_pa1_ value lower than this when they are in individual solutions (Table 2). In a previous study, ascorbic acid, caffeic acid, catechin, and hesperetin were better antioxidants in binary mixtures with quercetin [23]. 

There are six mixed solutions in which the E_pa1_ value is lower than the same for the two compounds in individual solutions: SYR-VAN (0.406 V), CAF-QUER (0.414 V), SYR-QUER (0.423 V), VAN-QUER (0.423 V), VAN-EPI (0.471 V), and EPI-RUT (0.471 V). Other studies indicated an oxidation potential lower for a mixture of quercetin and catechin than for both individual compounds [23]; however, in our work, this does not occur for quercetin.

**Figure 3:**
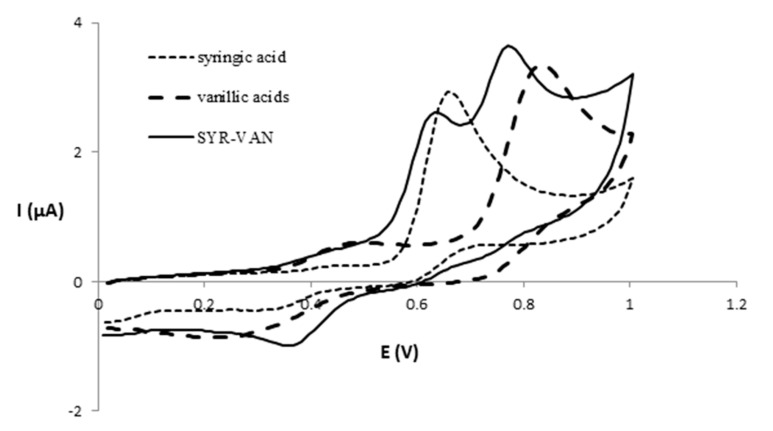
Voltammograms of syringic and vanillic acids for individual and binary model solutions.

Figure 3 shows the voltammograms corresponding to the syringic and vanillic acids in individual and binary model solutions (SYR-VAN). As can be seen, the binary model solution retained its two anodic peaks corresponding to the syringic and vanillic acids measured individually, and with the E_p_ values lower than those of the individual model solution. Thus, in the potential zone between 0.5 and 1 V, the E_pa_ values were 0.673 and 0.835 V for the syringic and vanillic acids, respectively, while in the SYR-VAN solution, two peaks appear at 0.633 and 0.770 V. This fact indicates a higher antioxidant activity for the binary model solution. 

A particular case is the mixed solution of caffeic acid and quercetin 3-*O*-rutinoside; here, the voltammogram does not show a second anodic peak related to quercetin 3-*O*-rutinoside. This could be due to the difference in I_pa_ values between these two compounds for the value of the corresponding potential (E_pa2_).

### 3.2. Electrochemistry of Amino Acids

CV was applied to monitor the electrochemical properties of amino acids, specifically cysteine, histidine, tyrosine, and tryptophan. First, the electrochemical conditions for cysteine were optimized. Then, under the optimized conditions, the electrochemical behavior of four individual model solutions and the electrochemical behavior of six binary model solutions (with two amino acids) were measured.

#### 3.2.1. Optimization of Electrochemical Conditions

Optimization conditions, such as pH (3.0 and 7.0) concentration (1.0 and 2.0 g/L), and scanning rate (100 and 50 mV/s) for the measurement of cyclic voltammetry, were studied in relation to cysteine. Table 3 shows the electrochemical parameters extracted from the cyclic voltammetry curve.

In the optimization of the electrochemical measurement conditions of cysteine, it was observed that only the different scanning rates show a significant effect (*p* < 0.05) in I_pa_ values. The voltammogram corresponding to cysteine at pH 7.0 shows two anodic peaks with E_pa_ values around 0.8 and 1.9 V. The first peak corresponds to the oxidation of the thiol group (-SH). Subsequent nucleophilic attack of water gives rise to the corresponding sulfinic acid. This process occurs at pH > 4 via the transfer of a proton and an electron [12]. The second anodic peak at around 1.9 V corresponded to the oxidation of sulfinic acid to cysteic acid. 

At pH 3.0, no anodic peaks appear in the voltammograms, except under conditions of 2.0 g/L for amino acids and 100 mV/s of scanning rate. The cathodic peak is not observed at any of the pH values studied, which confirms the irreversibility of the process. 

Taking into account the results obtained in the electrochemical properties of cysteine, the measurement conditions for the amino acid were pH 7.0, 2.0 g/L of concentration, and 100 mV/s of scanning rate.

#### 3.2.2. Electrochemistry of Amino Acids: Individual Model Solutions 

The electrochemical behavior of four amino acid compounds (cysteine, histidine, tyrosine, and tryptophan) has been evaluated under the previously established conditions. Table 4 shows the electrochemical parameters extracted from the voltammograms of the individual model solutions. 

In the voltammogram obtained for histidine, a single anodic peak appears at 1.482 V, corresponding to an oxidation process of the imidazole group that occurs only in the pH range 6 to 9. In the mechanism corresponding to this process, the transfer of a proton and an electron to form 2-oxo-histidine takes place [24]. The absence of any cathodic peak confirms the irreversibility of the process [24]. 

Specifically, tryptophan has a peak of oxidation at E_pa_ = 0.900 V. This anodic peak coincides with the oxidation of derivatives of the indole group with a substituent at the C3 position of the pyrrole ring [25].

In relation to tyrosine, its electrochemical behavior showed two peaks at E_pa1_ = 0.778 V and at E_pa2_ = 1.983 V. The oxidation peaks present in the tyrosine voltammogram are related to the para-substituted phenol present in its chemical structure [26]. The first peak is associated with the formation of the catechol group, and the second with the formation of the quinone group. 

According to the idea that a compound with a lower E value is a better antioxidant because it has a higher ability to donate electrons, the order E_pa1_ of amino acids is cysteine (0.754 V), tyrosine (0.778 V), tryptophan (0.900 V), and histidine (1.482 V).

#### 3.2.3. Electrochemistry of Amino Acids: Binary Model Solutions

The electrochemical behavior of the binary model solutions of amino acids was evaluated under conditions previously optimized for these compounds. A total of six binary model solutions were evaluated, considering all possible combinations with the amino acids studied: cysteine, histidine, tryptophan, and tyrosine. The results obtained are shown in Table 4. In the voltammograms obtained in the electrochemical measurements of the binary model amino acid solutions, peaks corresponding to each amino acid measured individually appear. These results agree with those obtained by Enache et al. (2017) [27], in which protein measurements give rise to voltammograms with characteristic peaks corresponding to the oxidizable amino acids that compose them. A particular case is that binary model solutions containing cysteine (CYS-HIS, CYS-TRP and TYR-CYS) show an anodic peak around E_pa3_ = 1.900 V corresponding to the oxidation of sulfinic acid to cysteic acid. 

As in the binary model solutions of phenolic compounds, the E_p_ values of the amino acid solutions were different for a compound alone or mixed. All binary model solutions of amino acids were a better antioxidant in combination than alone. In this regard, histidine was a better antioxidant combination (HIS: E_pa1_ = 1.482 V vs. CYS-HIS: E_pa1_ = 0.649 V, HIS-TRP: E_pa1_ = 0.722 V and TYR-HIS: E_pa1_ = 0.770 V). 

The binary model solutions with the lowest E_pa1_ values, and therefore the best antioxidants, are TYR-TRP (0.609 V), CYS-HIS (0.649 V), and CYS-TRP (0.706 V). 

With respect to the I_pa_ values, they are also affected in a binary model solution, as in the case of solutions of phenolic compounds, due to their relationship with the concentration.

### 3.3. Electrochemistry of Mixed Phenol–Amino Acid Solutions

For the evaluation of CV effectiveness in the evaluation of the synergistic effect of phenolic compounds and amino acids on antioxidant activity, mixed phenol–amino acid solutions were prepared. First, the pH of the mixed phenol–amino acid solutions was optimized due to differences in the pH of the individual model from phenolic compounds (pH 3.0) and amino acids (pH 7.0). In this regard, the CYS-CAF mixture solution was selected for this purpose.

The electrochemical behavior of this mixture at pH 3.0 showed one anodic peak, the first appearing at around 0.4 V and the second at 0.8 V. These values are very similar to the anodic peaks obtained when analyzing the components of the solution separately. On the other hand, at pH 7.0, two peaks are also obtained, but with a displacement of the E_pa_ value with respect to the individual measurements. At this pH, the peak corresponding to cysteine is also observed at 1.9 V, which is not seen at pH 3.0. This better behavior of amino acids at pH 7 allowed us to select this pH to carry out the measurement in mixed phenol–amino acid solutions.

For this, a total of 16 mixed phenol–amino acid solutions were evaluated considering all possible combinations, with two compounds of phenolic compounds (caffeic and gallic acids, catechin, and quercetin 3-*O*-rutinoside) and amino acids (cysteine, histidine, tryptophan, and tyrosine). The results obtained are shown in Table 5. As can be seen, there are many changes in the antioxidant behavior of individual model solutions vs. mixed phenol–amino acid solutions with electrolyte solution conditions of pH 7.0, 100 mV/s scanning rate and a concentration of 1 g/L for phenolic compounds and 2.0 g/L for amino acids. 

As can be observed in Table 5, in general, all mixed phenol–amino acid solutions showed E_pa1_ and E_pa2_. Additionally, cysteine solutions had E_pa4_ and histidine solutions had E_pa3_. 

To study the significant differences between the three types of solutions, an ANOVA was performed, considering the following parameters: E_pa1_, I_pa1_, E_pa2_, I_pa2_, E_pa3_, I_pa3_, E_pa4_, and I_pa4_. The electrochemical parameters E_pa1_ and I_pa1_ were found to be significant (*p* < 0.05), which indicates that the mixture of a phenolic compound and an amino acid produces changes in the electrochemical behavior relative to the individual compound. Specifically, this change is related to the individual model solutions of phenolic compounds.

In order to evaluate the synergistic effects of phenolic compounds and amino acids, the onset potentials (E_on_: potential where phenolic oxidation begins to occur) for the individual model solutions of phenolic compounds and for the mixed phenol–amino acid solutions were compared (Table 6). Additionally, I_pa_ values for the solutions were compared because increases in peak current for phenolic compounds upon the addition of an amino acid would be another indicator of synergistic effects. The electrochemical behavior was different depending on the compounds considered. 

**Figure 4:**
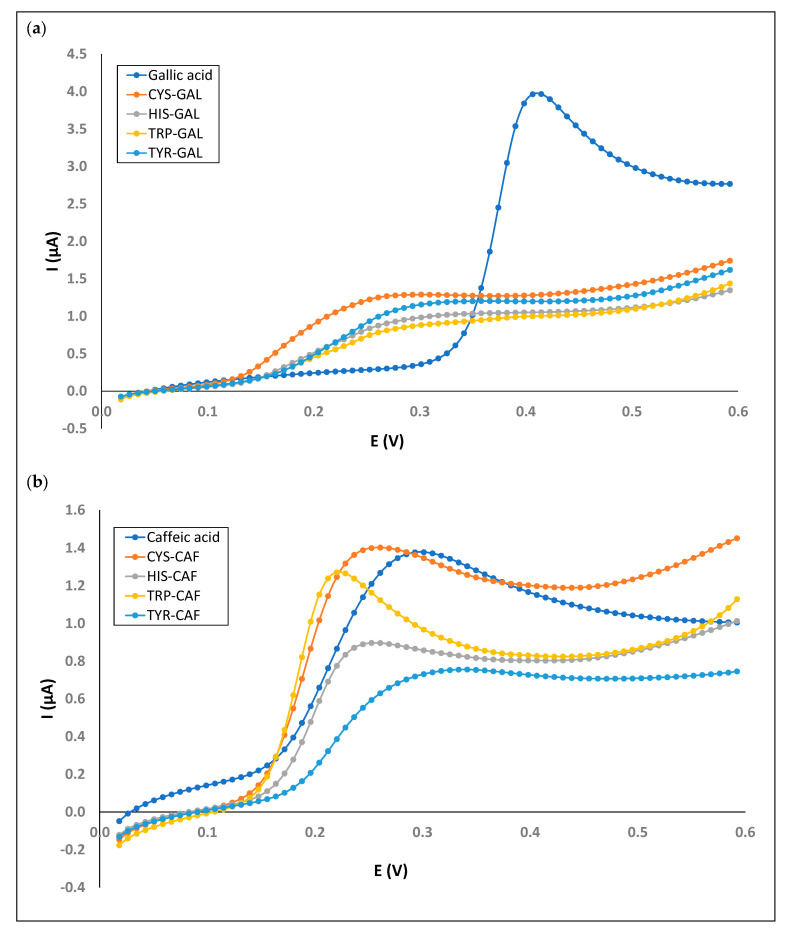
Comparation of voltammograms for individual model phenolic solutions (gallic acid (**a**) and caffeic acid (**b**)) and their associated mixed phenol–amino acid solutions.

Considering gallic acid, E_on_ for an individual model solution was 0.3255 V, while that of E_on_ was lower for all mixed solutions (between 0.1152 and 0.1233 V) (Figure 4a). The mixed solutions are then oxidized at a potential lower than that necessary to oxidize gallic acid directly. This fact indicates that there is a synergistic effect. However, I_pa_ decreases in all cases, indicating a lower quantitative effect. 

For caffeic acid, a positive synergistic effect considering both E_on_ and I_pa_ was observed in the mixed solution with cysteine. Additionally, a synergistic effect considering E_on_ was observed for a mixed solution with TRP (0.1395 V) (Figure 4b).

**Figure 5:**
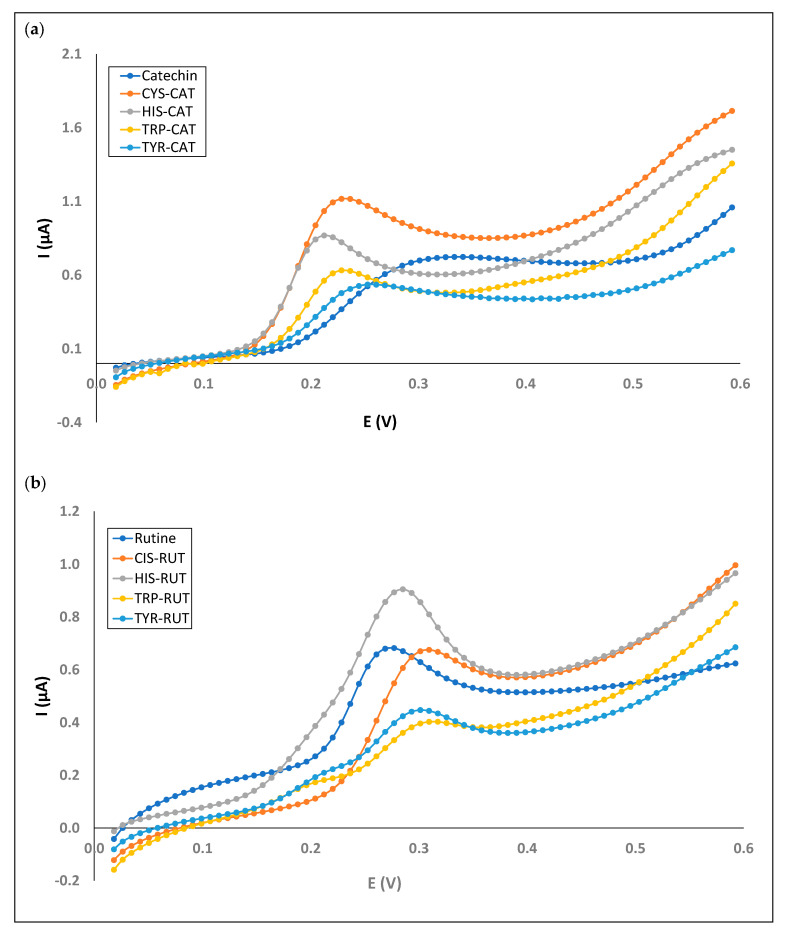
Comparation of voltammograms for individual model phenolic solutions (catechin (**a**) and rutine (**b**)) and their associated mixed phenol–amino acid solutions.

Regarding catechin, all of the mixed solutions had lower E_on_ than an individual solution (between 0.147 and 0.173 V vs. 0.179 V, respectively). In addition, mixed solutions with cysteine and histidine had increases in I_pa_ with respect to the individual solution (1.117 and 0.867 µA, respectively, vs. 0.723 µA) (Figure 5a). Considering quercetin 3-*O*-rutinoside, mixed solutions with HIS, TRP, and TYR are oxidized at a lower potential than individual solutions (0.179 V vs. 0.147, 0.115 and 0.115 V, respectively). The HIS-RUT solution showed a higher I_pa_ (0.905 µA) than the RUT solution (0.682 µA) (Figure 5b).

Considering both E_on_ and I_pa_ as electrochemical parameters to classify the synergistic effect, the mixed solutions CIS-CAT, HIS-CAT, CIS-CAF, and HIST-RUT had better effects.

## 4. Conclusions

Voltametric methods for the evaluation of the antioxidant activity of phenolic compounds and amino acids have been described, and their applicability is demonstrated by optimizing the experimental measurement conditions in model solutions. The synergistic effect of these compounds on antioxidant activity was determined, and the electrochemical parameter E_pa_ was successfully used to classify this activity into three types of model solutions (individual model solutions, binary model solutions, and mixed phenol–amino acid solutions). Additionally, the electrochemical parameter of E_on_ was used to evaluate the synergistic effects of phenolic compounds and amino acids.

The proposed electrochemical methods could be used in samples rich in phenolic compounds, amino acids, or a combination of both compounds. The results indicate that cyclic voltammetry is a suitable technique to evaluate the synergistic effect of phenolic and amino acid compounds on antioxidant activity.

## Figures and Tables

**Table 1:** Electrochemical parameters extracted from cyclic voltammetry curves in the optimization of measurement conditions for phenolic compounds (Significant differences (*p* < 0.05) are highlighted in italics: * pH, ** V, and *** C).

Compounds	pH	C (g/L)	V (mV/s)	E_pa1_ (V)	E_pc1_ (V)	I_pa1_ (μA)	I_pc1_ (μA)	E°_1_ (V)	ΔE_1_ (V)	E_pa2_ (V)	I_pa2_ (μA)
Gallic acid	3	1	100	0.414 *	0.350	3.965	−0.282	0.382 *	0.065 *	0.786 *	3.952
			50	0.414 *	0.350	2.160	0.603	0.382 *	0.065 *	0.786 *	2.209
		0.5	100	0.414 *	0.366	1.66	−0.254	0.390 *	0.048 *	0.803 *	1.900
			50	0.406 *	0.358	1.032	−0.162	0.382 *	0.048 *	0.786 *	1.097
	7	1	100	0.358 *	-	1.349	-	-	-	0.633 *	2.044
			50	0.350 *	-	1.024	-	-	-	0.641 *	1.402
		0.5	100	0.301 *	-	0.515	-	-	-	0.625 *	0.916
			50	0.350 *	-	0.428	-	-	-	0.641 *	0.620
Caffeic acid	3	1	100	0.439 *	0.309 *	1.802	−1.056 **	0.374 *	0.129	-	-
			50	0.423 *	0.334 *	1.122	−0.754 **	0.378 *	0.089	-	-
		0.5	100	0.447 *	0.334 *	1.717	−1.415 **	0.390 *	0.113	-	-
			50	0.431 *	0.350 *	1.079	−0.850 **	0.390 *	0.081	-	-
	7	1	100	0.301 *	0.099 *	1.471	−1.446 **	0.200 *	0.202	-	-
			50	0.309 *	0.083 *	0.820	−0.766 **	0.196 *	0.226	-	-
		0.5	100	0.261 *	0.131 *	0.831	−0.780 **	0.196 *	0.129	-	-
			50	0.253 *	0.139 *	0.544	−0.526 **	0.196 *	0.113	-	-
Catechin	3	1	100	0.495 *	0.261 *	1.693	−1.209	0.378 *	0.235 ***	0.835	2.355
			50	0.479 *	0.285 *	0.867	−0.625	0.382 *	0.194 ***	0.843	1.218
		0.5	100	0.447 *	0.325 *	1.481	−1.374	0.386 *	0.121 ***	0.811	1.111
			50	0.487 *	0.301 *	0.612	−0.533	0.394 *	0.186 ***	0.827	0.692
	7	1	100	0.334 *	0.107 *	0.723	−0.540	0.220 *	0.226 ***	0.681	1.371
			50	0.342 *	0.107 *	0.311	−0.286	0.224 *	0.235 ***	0.706	0.692
		0.5	100	0.293 *	0.131 *	0.705	−0.703	0.212 *	0.162 ***	0.641	0.800
			50	0.293 *	0.123 *	0.403	−0.379	0.208 *	0.170 ***	0.673	0.494
Quercetin−3-*O*-rutinoside	3	1	100	0.479 *	0.431 *	0.965	−0.517	0.455 *	0.049	0.754	0.860
			50	0.479 *	0.431 *	0.648	−0.344	0.455 *	0.049	0.730	0.542
		0.5	100	0.487 *	0.309 *	0.969	−0.583	0.398 *	0.178	-	-
			50	0.479 *	0.325 *	0.507	−0.336	0.402 *	0.154	-	-
	7	1	100	0.277 *	0.212 *	0.682	−0.419	0.245 *	0.065	0.867	1.555
			50	0.277 *	0.220 *	0.415	−0.251	0.249 *	0.057	0.867	1.103
		0.5	100	0.301 *	*-*	0.465	-	-	-	0.916	1.000
			50	0.301 *	-	0.226	-	-	-	0.916	0.545

**Table 2:** Electrochemical behavior of phenolic compounds under optimized conditions (pH 3.0; 1.0 g/L and 100 mV/s).

Compounds	E_pa1_ (V)	E_pc1_ (V)	I_pa1_ (μA)	I_pc1_ (μA)	E°_1_ (V)	ΔE_1_ (V)	E_pa2_ (V)	I_pa2_ (μA)	E_pa3_ (V)	I_pa3_ (μA)
Individual Model solutions										
Gallic acid	0.414	0.350	3.965	−0.282	0.382	0.065	0.786	3.952	-	-
Syringic acid	0.447	0.333	0.243	−0.436	0.390	0.114	0.673	2.931	-	-
Vanillic acid	0.487	0.228	0.594	−0.867	0.358	0.259	0.835	3.359	-	-
Caffeic acid	0.439	0.309	1.802	−1.056	0.374	0.129	-	-	-	-
Ferulic acid	0.471	0.269	0.629	−0.997	0.370	0.202	0.754	2.418	-	-
Catechin	0.495	0.261	1.693	−1.209	0.378	0.235	0.835	2.355		
Epicatechin	0.495	0.148	1.146	−0.694	0.321	0.348	0.851	1.931	-	-
Quercetin 3-rutinoside	0.479	0.431	0.965	−0.517	0.455	0.049	0.754	0.860	-	-
Quercetin	0.439	0.228	1.733	−0.856	0.334	0.210	0.851	1.684	-	-
Binary model solutions ^1^										
CAF-RUT	0.479	0.269	1.344	−0.757	0.374	0.210	-	-	-	-
CAT-CAF	0.511	0.212	1.269	−0.823	0.362	0.299	0.859	1.474	-	-
CAT-RUT	0.592	0.245	0.799	−0.514	0.418	0.348	0.883	1.371	-	-
CAT-EPICAT	0.552	0.115	1.212	−0.673	0.334	0.437	0.867	2.074	-	-
FER-CAF	0.511	0.212	1.592	−1.135	0.362	0.299	0.722	2.046	-	-
GAL-SYR	0.552	0.164	1.297	−0.427	0.358	0.388	0.706	2.030	-	-
GAL-VAN	0.552	-	1.318	-	-	-	0.851	2.550	-	-
CAF-EPICAT	0.455	0.285	1.694	−1.033	0.370	0.170	0.819	1.810	-	-
CAF-SYR	0.455	0.309	1.468	−0.975	0.382	0.146	0.665	2.376	-	-
CAF-VAN	0.495	0.220	1.277	−0.896	0.358	0.275	0.835	2.351	-	-
FER-CAT	0.536	0.204	1.087	−0.968	0.370	0.332	0.835	2.249	-	-
FER-EPICAT	0.503	0.172	0.896	−0.778	0.338	0.332	0.835	2.127	-	-
FER-GAL	0.439	0.334	1.823	−0.505	0.386	0.105	0.633	2.716	0.738	3.026
FER-QUER	0.439	0.196	1.458	−1.078	0.317	0.243	0.851	2.148	-	-
FER-RUT	0.479	0.301	0.788	−0.726	0.390	0.178	0.730	1.723	-	-
GAL-EPICAT	0.439	0.342	2.428	−0.724	0.390	0.097	0.762	3.014	-	-
GAL-QUER	0.536	0.293	2.313	−0.821	0.414	0.243	0.843	2.856	-	-
CAT-QUER	0.463	0.293	2.208	−1.282	0.378	0.170	0.859	3.288	-	-
EPICAT-QUER	0.455	0.261	2.061	−1.139	0.358	0.194	0.851	3.021	-	-
EPICAT-RUT	0.471	0.293	1.826	−1.080	0.382	0.178	0.811	2.104	-	-
SYR-CAT	0.479	0.277	1.208	−1.040	0.378	0.202	0.657	2.272	0.843	2.212
SYR-EPICAT	0.487	0.228	1.104	−0.881	0.358	0.259	0.673	2.131	0.827	2.120
SYR-QUER	0.423	0.237	1.481	−0.829	0.330	0.186	0.859	2.049	-	-
SYR-RUT	0.479	0.228	0.842	−0.570	0.354	0.251	0.681	1.550	-	-
SYR-VAN	0.406	0.358	0.411	−0.987	0.382	0.049	0.633	2.620	0.770	3.638
VAN-CAT	0.487	0.253	1.098	−1.296	0.370	0.235	0.795	4.771	-	-
VAN-EPICAT	0.471	0.261	1.175	−1.193	0.366	0.210	0.803	3.275	-	-
VAN-QUER	0.423	0.196	1.659	−0.992	0.309	0.226	0.843	3.092	-	-
FER-VAN	0.479	0.261	0.529	−0.960	0.370	0.218	0.641	1.435	0.811	2.896
CAF-QUER	0.414	0.212	1.559	−1.239	0.313	0.202	0.544	2.108	0.875	2.029
RUT-QUER	0.463	0.293	1.565	−0.740	0.378	0.170	0.851	1.689	-	-
CAF-GAL	0.463	0.325	2.465	−0.610	0.394	0.137	0.795	1.830	-	-
FER-SYR	0.495	0.293	0.604	−0.759	0.394	0.202	0.673	2.480	0.754	2.415
GAL-CAT	0.487	0.293	1.947	−0.535	0.390	0.194	0.843	2.201	-	-
GAL-RUT	0.487	0.366	1.820	−0.206	0.427	0.121	0.778	1.607	-	-
VAN-RUT	0.479	0.423	0.947	−0.675	0.451	0.057	0.786	2.825	-	-

^1^ GAL: gallic acid; SYR: syringic acid; VAN: vanillic acid; CAF: caffeic acid; FER: ferulic acid; CAT: catechin; EPICAT: epicatechin; RUT: quercetin-3-*O*-rutinoside; QUER: quercetin.

**Table 3:** Electrochemical parameters extracted from the cyclic voltammetry curves in the optimization of cysteine.

Compounds	pH	C (mM)	V (mV/s)	E_pa1_ (V)	I_pa1_ (μA)	E_pa2_ (V)	I_pa2_ (μA)
Cysteine	3	2.5	100	-	-	-	-
			50	-	-	-	-
		5.0	100	0.487	0.216	-	-
			50	-	-	-	-
	7	2.5	100	0.891	0.990	1.847	22.034
			50	0.843	0.495	1.842	18.139
		5.0	100	0.802	1.072	1.918	167.449
			50	0.831	0.520	1.886	149.261

**Table 4:** Electrochemical behavior of cysteine, histidine, tyrosine, and tryptophan under optimization conditions (pH 7.0; 2.0 g/L and 100 mV/s).

Compounds	E_pa1_ (V)	I_pa1_ (μA)	E_pa2_ (V)	I_pa2_ (μA)	E_pa3_ (V)	I_pa3_ (μA)
Individual Model solutions						
Cysteine	0.754	0.767	-	-	1.918	167.449
Histidine	-	-	1.482	22.617	-	-
Tryptophan	0.900	2.254	-	-	-	-
Tyrosine	0.778	1.481	-	-	1.983	201.152
Binary model solutions ^1^						
CYS-HIS	0.649	2.396	1.231	20.947	1.911	169.067
CYS-TRP	0.706	3.054	-	-	1.854	137.868
HIS-TRP	0.722	2.435	1.336	19.942	-	-
TYR-CYS	0.754	1.805	-	-	1.967	194.895
TYR-HIS	0.770	0.939	1.369	14.276	-	-
TYR-TRP	0.609	0.316	1.094	1.230	-	-

^1^ CYS: cysteine; HIS: histidine; TRP: tryptophan; TYR: tyrosine.

**Table 5:** Electrochemical behavior of individual model solutions and mixed phenol–amino acids solutions at pH 7.0, 100 mv/S and concentration of 1.0 g/L for phenolic compounds and 2.0 g/L for amino acids.

	E_pa1_ (V) ^1^	I_pa1_ (μA)	E_pa2_ (V) ^1^	I_pa2_ (μA)	E_pa3_ (V) ^1^	I_pa3_ (μA)	E_pa4_ (V) ^1^	I_pa4_ (μA)
Individual Model solutions								
Gallic acid	0.358	1.349	0.633	2.044	-	-	-	-
Caffeic acid	0.301	1.471	-	-	-	-	-	-
Catechin	0.334	0.723	0.681	1.371	-	-	-	-
Quercetin 3-*O*-rutinoside	0.277	0.682	0.867	1.555	-	-	-	-
Cysteine	-	-	0.802	1.072	-	-	1.918	167.449
Histidine	-	-	-	-	1.482	22.617	-	-
Tryptophan	-	-	0.900	2.254	-	-	-	-
Tyrosine	-	-	0.778	1.481	-	-	1.983	201.152
Mixed Solutions ^2^								
CYS-CAF	0.261	1.402	0.697	3.312	-	-	1.911	170.227
CYS-CAT	0.228	1.117	0.665	1.968	-	-	1.911	157.796
CYS-GAL	0.301	1.288	0.722	2.129	-	-	1.902	90.454
CYS-RUT	0.309	0.676	0.924	3.101	-	-	1.967	166.534
HIS-CAF	0.253	0.896			1.288	16.786	-	-
HIS-CAT	0.212	0.868	0.592	1.450	1.377	23.887	-	-
HIS-GAL	0.358	1.039	0.665	1.571	1.344	20.235	-	-
HIS-RUT	0.285	0.905	0.916	3.132	1.417	24.368	-	-
TRP-CAF	0.220	1.271	0.697	3.312	-	-	-	-
TRP-CAT	0.228	0.631	0.722	2.639	-	-	-	-
TRP-GAL	0.269	0.811	0.714	3.039	-	-	-	-
TRP-RUT	0.317	0.403	0.714	2.451	-	-	-	-
TYR-CAF	0.342	0.756	0.786	1.100	-	-	-	-
TYR-CAT	0.253	0.536	0.673	0.998	-	-	-	-
TYR-GAL	0.358	1.202	0.746	2.090	-	-	-	-
TYR-RUT	0.301	0.447	0.770	1.293	0.932	2.179	-	-

^1^ Epa_1_ = 0–0.5 V, Epa_2_ = 0.5–0.95 V, Epa_3_ = 0.95–1.5 V, Epa_4_ = 1.5–2.0 V. ^2^ GAL: gallic acid; CAF: caffeic acid; CAT: catechin; RUT: quercetin 3-*O*-rutinoside; CYS: cysteine; HIS: histidine; TRP: tryptophan; TYR: tyrosine.

**Table 6:** Comparation of values of E_on_ and I_pa_ for individual model phenolic solutions and their associated mixed phenol–amino acid solutions.

Solution ^1^	E_on_ (V)	I_pa_ (μA)
Gallic acid	0.325	3.962
CIS-GAL	0.115	1.277
HIS-GAL	0.123	1.025
TRP-GAL	0.123	0.920
TYR-GAL	0.123	1.194
Caffeic acid	0.155	1.378
CIS-CAF	0.147	1.402
HIS-CAF	0.161	0.896
TRP-CAF	0.139	1.270
TYR-CAF	0.171	0.752
Catechine	0.179	0.723
CIS-CAT	0.147	1.117
HIS-CAT	0.147	0.867
TRP-CAT	0.163	0.631
TYR-CAT	0.173	0.535
Quercetin 3-*O*-rutinoside	0.179	0.682
CIS-RUT	0.220	0.675
HIS-RUT	0.147	0.905
TRP-RUT	0.115	0.402
TYR-RUT	0.115	0.447

^1^ GAL: gallic acid; CAF: caffeic acid; CAT: catechin; RUT: quercetin 3-*O*-rutinoside; CYS: cysteine; HIS: histidine; TRP: tryptophan; TYR: tyrosine.

## Data Availability

The original contributions presented in the study are included in the article, further inquiries can be directed to the corresponding author.
